# Hybrid Strategy for Residual Arch and Thoracic Aortic Dissection following Acute Type A Aortic Dissection Repair

**DOI:** 10.1155/2014/165425

**Published:** 2014-02-19

**Authors:** Sidharth Viswanathan, Vivek Agrawal, Shashidhar Kallappa Parameshwarappa, Ajay Savlania, Santhosh Kumar, Unnikrishnan Madathipat

**Affiliations:** ^1^Division of Vascular and Endovascular Surgery, Department of Cardio-Thoracic and Vascular Surgery, Sree Chitra Tirunal Institute for Medical Sciences and Technology, Trivandrum, Kerala 695011, India; ^2^Department of Imaging Sciences and Interventional Radiology, Sree Chitra Tirunal Institute for Medical Sciences and Technology, Trivandrum, Kerala 695011, India

## Abstract

Progressive dilatation of the false lumen in the arch and descending aorta has been encountered in one-third of survivors as a late sequelae following repair of ascending aortic dissection. Conventional treatment for the same requiring cardiopulmonary bypass and deep hypothermic circulatory arrest is associated with high morbidity and mortality especially in the elderly cohort of patients. Herein we report a case of symptomatic progressive aneurysmal dilatation of residual arch and descending thoracic aortic dissection following repair of type A aortic dissection, successfully treated by total arch debranching and ascending aortic prosthesis to bicarotid and left subclavian bypass followed by staged retrograde aortic stent-graft deployment. This case report with relevant review of the literature highlights this clinical entity and the present evidence on its appropriate management strategies. Close surveillance is mandatory following surgical repair of type A aortic dissection and hybrid endovascular procedures seem to be the most dependable modality for salvage of patients detected to have progression of residual arch dissection.

## 1. Introduction

Dissection of the ascending aorta is a well-recognized medical emergency that warrants urgent surgical repair to save life. This involves replacement of the ascending aorta and reconstruction of aortic root to restore aortic valve competence by repair or replacement, with intent to provide exclusive true lumen flow distally. Progressive dilatation of the false lumen in the arch and descending aorta has been encountered in one-third of survivors as late sequelae following repair of ascending aortic dissection [1]. Conventional treatment of this delayed complication involves open arch repair with elephant trunk procedure requiring cardiopulmonary bypass (CPB) and deep hypothermic circulatory arrest (DHCA) with its inherent high morbidity and mortality. Hybrid endovascular therapy is fast evolving as the current standard of care for extensive thoracic aortic diseases. This case report with literature review highlights the clinical entity of residual arch and descending aortic dissection following surgical repair of type A aortic dissection and its effective treatment by hybrid thoracic endovascular aortic repair (TEVAR).

## 2. Case Presentation

**Figure 1:**
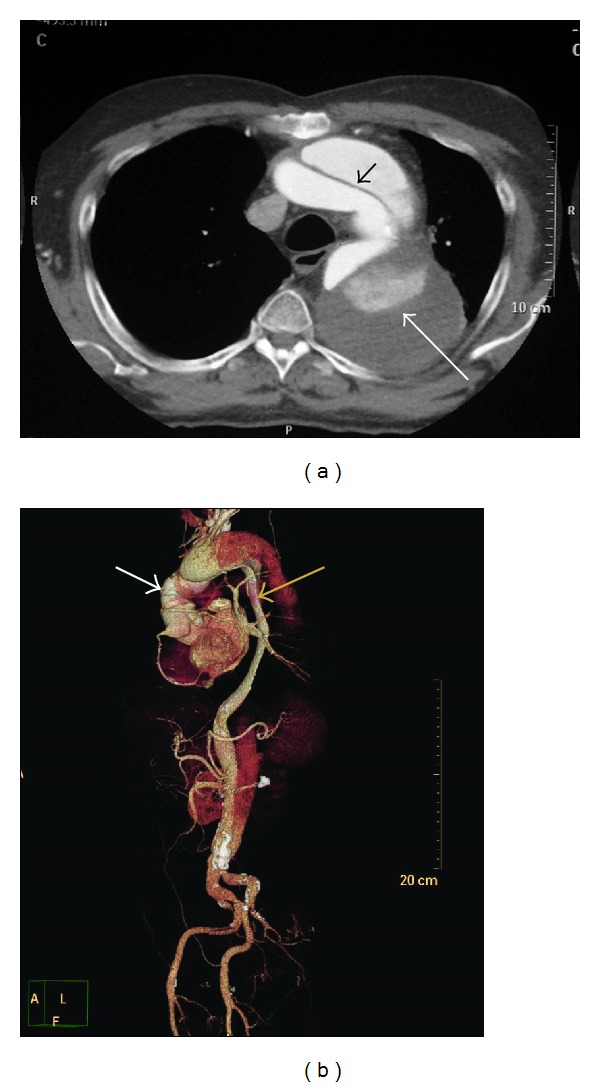
(a) Preoperative axial contrast-enhanced CT chest showing intimomedial flap in the aortic arch (black arrow) and partially thrombosed aneurysmal false lumen (white arrow). (b) CT angiogram, volume-rendered 3D image showing residual aortic arch and descending aortic dissection with aneurysmal degeneration (black arrow) following prior graft replacement of ascending aorta (white arrow) for type A aortic dissection.

**Figure 2:**
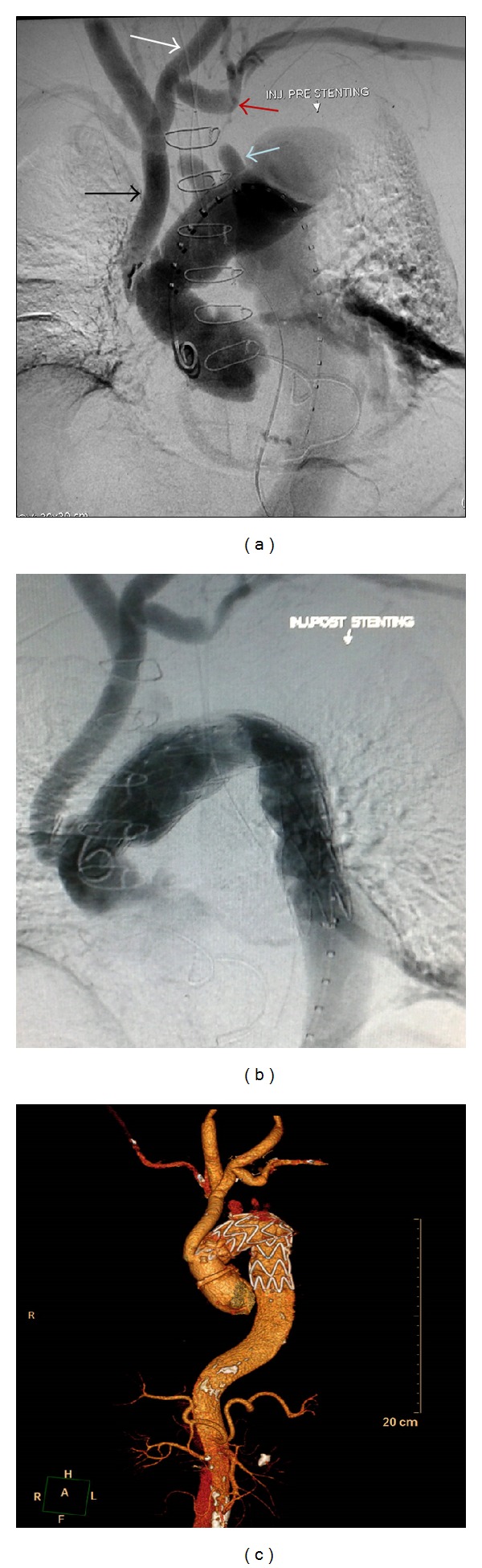
(a) Digital subtraction angiogram prior to stent graft deployment showing patent grafts taking inflow from previously placed ascending aortic graft with 10 mm graft to left common carotid artery (black arrow), piggybacking an 8 mm graft to left common carotid artery (white arrow), and another 8 mm graft to left subclavian artery (red arrow). The native stumps of the debranched arch vessels are also seen (blue arrow). (b) Check angiogram after stent graft deployment showing opened-up true lumen and nonopacification of false lumen. (c) CT angiogram, volume-rendered 3D image at 1 year follow-up showing patent bypass grafts, significantly remodelled aorta, and near-total thrombosis of false lumen.

67-years-old retired professor, known hypertensive for 12 years and reformed smoker, underwent repair of type A aortic dissection 4 years back by supracoronary ascending aortic replacement with aortic valve resuspension. He later presented with worsening dyspnoea and left sided chest pain over the past 1 year. On evaluation he had lower blood pressure on left upper limb as compared to the right with gradient of 20 mmHg. All peripheral pulses were palpable. Laboratory parameters, electrocardiogram, and echocardiogram were normal. CT angiogram revealed patent mid ascending aortic prosthetic graft with native aortic root and normal coronaries, with dissection flap noted in the arch (Figure 1(a)) and descending aorta extending across the abdominal aorta up to the bifurcation of right common iliac artery (Figure 1(b)). Innominate, left common carotid, celiac, superior mesenteric, and right renal arteries were arising from true lumen. Flap was noted extending into left subclavian artery (SCA) and left renal artery with hypoperfusion of left kidney. Aneurysmal false lumen measured a maximum diameter of 73 mm in upper descending thoracic aorta (DTA). Debranching of the arch vessels was undertaken as 1st stage of the hybrid repair using a 10 mm knitted bovine gelatin coated polyester graft (Uni-graft K DV, Braun Aesculap, Tuttlingen, Germany) for bypass from ascending aortic prosthesis to the right common carotid artery, piggybacking 8 mm grafts to left common carotid artery and left subclavian artery (Figure 2(a)). In view of redo sternotomy, femoral vessels and right axillary artery were kept exposed in preparation for CPB although the need did not arise. He tolerated the procedure well except for acute exacerbation of bronchospasm in the postoperative period that settled with medications. On recovery endorepair was accomplished by deployment of 36 × 200 mm Valiant Thoracic Captivia aortic stent graft (Medtronic Inc., Minneapolis, MN, USA) from within the distal ascending aortic prosthetic graft across the debranched arch up to mid DTA, confirming nonopacification of false lumen on check angiogram (Figure 2(b)). His postprocedure period was uneventful except for lymphorrhoea from groin wounds that settled on conservative management. At 1 year follow-up he remains symptom free and active, with CT angiogram showing patent bypass grafts, significantly remodelled aorta, and near-total thrombosis of false lumen (Figure 2(c)).

## 3. Discussion

Since Morgagni's first comprehensive description in 1761, aortic dissection has continued to pose great therapeutic challenge. Due to the high fatality risk associated with type A dissections, secondary to adventitial rupture causing cardiac tamponade or massive bleeding into pleural cavity, coronary disruption or severe aortic valvular regurgitation, emergent surgery, is mandatory at diagnosis [2].

Despite successful surgical repair, a residual dissection flap with enlarging false lumen may persist in the arch and descending thoracic aorta (DTA) requiring reintervention in 25–40% of patients [3, 4]. The main indications for reoperation are progressive enlargement of the false lumen of the aortic arch or descending aorta and suture line dehiscence [5]. Surgical repair of such residual dissections is technically demanding, requiring complex circulatory management, and carries high risk for preoperative cardiac, neurological, and other systemic complications. Although the results of total arch repair have improved in the recent years, the morbidity and mortality associated with conventional repair using CPB and DHCA are not insignificant [6]. Over the last decade, various strategies have been employed to decrease the risk of total arch replacement. Open ascending aortic and arch repair with a “frozen” elephant trunk technique using stent graft for the descending aorta have been developed and advocated [7]. Although these approaches have been shown to promote false lumen thrombosis and may decrease long-term complications, morbidity and mortality rates remain substantial.

Endovascular stent graft placement has evolved into an effective treatment modality of various disease states of the aorta, particularly in the complex anatomic regions of the arch extending to DTA. This technology continues to evolve at a rapid pace, leading to its wide acceptance. The major advantage of this approach is the avoidance of DHCA. This technique requires suitable “landing zones” (minimum 2 cm of “normal” aortic segment) for secure stent graft fixation, which in our case was the ascending aortic prosthetic graft. Surgical bypass grafting of the supra-aortic trunks provides the extended proximal landing zone to facilitate optimal stent graft apposition. The stent graft deployment may be done synchronous with the debranching procedure including the option of antegrade deployment through a side graft attached to ascending aortic graft although many surgeons consider performing them in a staged manner to lessen the magnitude of physiological stress to the patient [8], as was done in the present case. Controversy exists over the management of left subclavian artery (LSA) in such cases. While a few authors have recommended safe intentional coverage without revascularization [9], others have shown higher incidence of neurological sequelae and arm ischemia if no bypass was given to LSA [10]. LSA revascularisation is recommended in specific instances if stenoses and abnormalities of the supra-aortic and intracranial arteries are present or entire descending thoracic aorta needs to be relined.

Multiple risk factors have been elucidated for progressive patency and dilatation of false lumen following type A aortic dissection repair. These include uncontrolled hypertension, total size of DTA >35 mm at initial presentation [11], complete or partial patency of false lumen with a diameter >22 mm [12], and a large entry tear of more than 10 mm [13]. Experience with surgical bypass grafting in conjunction with TEVAR in a patient subsequent to ascending aortic replacement for type A dissection is growing [14]. Considering the difficulty and challenge in redo procedures on the ascending aorta, some authors have advocated prior surgical debranching (and bypass) of the innominate artery at the time of ascending aortic repair so that if the need arises, the hybrid endovascular procedure can be accomplished by an extrathoracic approach in the neck through a carotid-carotid bypass with or without a carotid-subclavian bypass with limited risk to the patient [15]. Such an option may be considered during the initial operation if the above mentioned risk factors are present. Yet another alternative is to complete the hybrid arch repair following the ascending aortic graft replacement by total arch debranching with antegrade stent graft deployment in a single stage. Sequential clamping of carotid arteries while debranching, although for a short period, coupled with DHCA could lead to significant adverse cerebral events and hence is not a preferred option. In the near future, fenestrated and branched grafts may obviate the need for hybrid procedures [16]. However, clinical trials of these grafts are yet to conclusively prove the safety and long-term efficacy of these devices. As experience with deploying stents in the proximal aorta increases, isolated endovascular repair of type A dissection may become a reality for patients previously considered too high risk for surgery [17]. Major concerns with these procedures still center on wire manipulation in these arches with large atherosclerotic burden without distal protection strategies. Presently, hybrid total arch repair offers debranching and bypass of the arch vessels (potential neuroembolic protection) prior to any manipulation in the arch.

Hybrid techniques certainly constitute an effective strategy in patients who are elderly with significant comorbidities unfavourable for open surgical repair, although the long-term results in terms of reoperations or reinterventions are still awaited. One concern would be progressive dilatation of the proximal or distal seal zones of the stent graft which might lead to lack of apposition and subsequent perfusion of the excluded aneurysm sac particularly in the context of dissection, mainly pertaining to the stability of these stent grafts in the acutely curved aortic arch domain [18]. Nevertheless, endovascular strategy helps bring to the forefront a state-of-the-art and innovative approach to the extensively diseased arch and thoracic aorta with more predictable success in treating these otherwise morbid and elderly patients, currently at midterm in our experience.

## 4. Conclusion

Close follow-up is mandatory in patients following open surgical repair for type A aortic dissection for detecting progression of residual dissection into the arch and descending thoracic aorta. A focused and individualised approach to the aortic arch at the initial surgery may be justified in those patients that are at risk for such complications. Hybrid repair has the potential to reduce operative morbidity and mortality as compared to formal surgical total arch replacement and needs to be studied for their potential impact on long-term outcomes.
